# Parathyroidectomy: is vitamin D a player for a good outcome?

**Published:** 2016

**Authors:** M Carsote, DN Paduraru, AE Nica, A Valea

**Affiliations:** * Department of Endocrinology, ”Carol Davila” University of Medicine and Pharmacy, Bucharest, Romania; “C. I. Parhon” National Institute of Endocrinology, Bucharest, Romania; ** Department of Surgery, ”Carol Davila” University of Medicine and Pharmacy, Bucharest, Romania; University Emergency Hospital, Bucharest, Romania; *** Department of Anesthesiology, ”Carol Davila” University of Medicine and Pharmacy, Bucharest, Romania; University Emergency Hospital, Bucharest, Romania; **** Department of Endocrinology, ”I. Hatieganu” University of Medicine and Pharmacy, Cluj-Napoca, Romania; Clinical County Hospital, Cluj-Napoca, Romania

**Keywords:** parathyroidectomy, vitamin D, primary hyperparathyroidism

## Abstract

**Background**:

The field of parathyroidectomy (PTx) is complex and brings together many specialists. Even if the surgical approaches changed from classical to minimally invasive PTx, a good outcome is correlated with an adequate localization before and during PTx, while blood assays, such as parathormone (PTH) or 25-hydroxyvitamin D, become useful additional markers.

**Aim.** Specific aspects related to parathyroidectomy and vitamins D (VD) were introduced.

**Material and Method.** The article represents a PubMed-based narrative review.

**Results.** The growing evidence regarding the high prevalence of hypovitaminosis D and early detection of primary hyperparathyroidism (HPT) requires a particular attention to the association of these two disorders, which may be incidental, but some common pathogenic links are displayed. Low VD stimulates PTH production as a secondary or even tertiary type of HPT diagnosis. VD deficiency is associated with larger parathyroid adenomas and higher levels of PTH before and after surgery for primary HPT. Asymptomatically and normocalcemic forms of primary HPT, which are not immediately referred to PTx, require a normalization of the VD levels. VD supplements are safe under some serum calcium cutoffs and offer a better outcome after PTx. However, primary HPT is cured by surgery and, if the indication is well established, this should not be delayed too long to replace VD. Up to half of PTx cases may experience increased PTH levels after surgery, but most of these are transitory if rapid VD correction is done and only a few remaining cases will eventually develop persistent / recurrent primary HPT.

**Conclusion.** A close following of 25-hydroxivitamin D represents one of the keys for a good outcome in the field of parathyroid surgery.

**Abbreviations**:

HPT = hyperparathyroidism, MEN = Multiple Endocrine Neoplasia Syndrome, PTx = parathyroidectomy, PTH = parathormone, VD = Vitamin D.

## Background

The field of parathyroidectomy (PTx) is complex and it brings together different specialists starting with endocrinologists who first establish the diagnosis of primary hyperparathyroidism (HPT) and then collaborate with the imagery team to have an adequate localization of the parathormone (PTH) excess source[**1**]. Once the removal of the parathyroid adenoma or hyperplasic glands is needed, a surgery and anesthesia team will be necessary [**2**]. Further on, the postoperative outcome needs endocrinology surveillance of the calcium and PTH levels variations because of the risk of hypoparathyroidism, on one hand, or, on the other hand, persistent/recurrent HPT, which may be found as specific endocrine anomalies of the parathyroid [**3**]. In cases with genetic backgrounds, such as Multiple Endocrine Neoplasia (MEN) Syndrome (type I or II), the thyroid or adrenal tumor diseases will eventually require specific surgery [**4**-**6**].The indications of parathyroidectomy vary, like the presence of high calcium levels, secondary osteoporosis, kidney stones to young patients’ age [**7**,**8**]. Even if the surgical approaches changed from classical to minimally invasive PTx, the key for a good outcome is an adequate localization before and during PTx, while blood assays such as PTH or 25-hydroxyvitamin D become useful additional markers [**9**,**10**].

## Aim

Specific aspects related to parathyroidectomy and vitamin D are introduced, focusing on a direct connection between VD and primary HPT and the way this affects the outcome after PTx unless pre-operative VD ranges are not optimal.

## Material and Method

The article represents a narrative review based on an English-language PubMed research of the medical and surgical literature. 

## Results

***Vitamin D: direct effects on primary HPT***

VD has specific receptors at the level of PTH producing parathyroid cells, displaying a direct inhibitor effect regarding hormonal secretion [**11**]. Based on a regular feedback mechanism, the VD deficiency stimulates the cells causing secondary HPT, which is a common fact in some geographical areas with low sun exposure, in the elderly communities or in cases with renal failure (so-called renal HPT) [**12**-**14**].Recent studies pointed the fact that in patients with primary HPT, a secondary component of high PTH levels is brought by vitamin D deficiency, regardless of its cause or seasonal anomalies [**15**,**16**].If low levels of VD are not corrected before PTx, a part from higher levels of PTH, parathyroid adenomas seem larger while the phenotype in primary HPT is more severe, especially regarding bone involvement [**17**-**19**].In order to supplement vitamin D before PTx,the exact thresholds of 25-hydroxivitamin D are not very well established (currently, the recommendation is to supplement VD if 25-hydroxivitamin D is less than 20 ng/mL) [**20**-**22**].Moreover, intra-operative PTH kinetics after PTx is not changed if VD deficiency is not corrected but PTH is displayed at higher values [**23**,**24**]. Thus, hypovitaminosis D therapy,such as daily oral supplements before PTx, will prevent the development of post-operatory normocalcemic HPT, which is found in up to 43% of the cases in some studies [**25**].However, other factors have been found in correlation with the lack of PTH normalization after surgery, a part from the VD levels, one of them being the correct identification and further removal of the adenoma; for instance, a six-years study showed that focused-approached surgery had higher PTH levels two weeks after PTx compared to the four-gland exploration [**26**].The other parameters like total blood calcium, parathormone, bone turnover markers such as alkaline phosphatase,which are higher on patients with primary HPT and severe VD insufficiency, have been studied, while one of the best prognosis tools remains the intra-operative morning PTH, being correlated with the post-operatory eucalcemic state more accurately than the VD status itself (based on some observations) [**27**].Overall, an elevated PTH value is expected after PTx (the percents depend on study from 9% to 62%), but this is not necessarily the failure of surgery,however, it may be an uncorrected VD deficiency, hungry bone syndrome in conditions with a long medical history before PTx or, exceptionally, a PTH resistance or reduced peripheral sensitivity to the hormone, or even unrecognized mild chronic kidney disease with inadequate function [**28**]. These causes are easy to adjust if they are correctly identified, mostly by VD supplementation (probably,except for the renal damage, but the VD replacement is carefully needed in this particular context, too) [**29**,**30**].Most of these situations with elevated PTH will eventually correct while a small number of the remaining cases will need a close check-up since a persistent or recurrent HPT should be assessed [**31**-**33**]. 

***Vitamin D deficiency before PTx***

VD deficiency is largely known and an incidental overlap with primary HPT might be expected since both of the conditions have been recently widely recognized and early detected due to accessible assays (the most affected population being menopausal women) [**34**,**35**]. The importance of VD supplementation before parathyroid surgery is still a matter of debate and many clinicians are actually afraid of offering VD replacement to a patient who probably already has a high calcium level but the VD replacement is actually safe in most situations (with a calcium level below 3 mmol/L) [**36**,**37**]. Some observations showed that in primary HPT the conversion of 25-hydroxyvitamin D to a 24-hydroxylated component is increased, thus, the prevalence of VD deficiency might be even higher [**37**].But, on the contrary, a long standing severe VD deficiency hyper-stimulates parathyroid glands to produce PTH on an autonomic basis as seen in tertiary HPT [**38**].Inadequately low VD means an additional PTH increase and a more aggressive phenotype of primary HPT [**39**].Even if the correction of VD insufficiency in mild forms of primary HPT will not exacerbate hypercalcemia or hypercalciuria and will not eventually cure the PTH-producing tumor as PTx does, some authors consider that the adenoma excision should not be delayed in order to restore the VD pool [**39**-**41**]. However, not all the cases of primary HPT are referred from the start to surgery, especially asymptomatically and normocalcemic variants and these particular situations will benefit from the VD supplementation when 25-hydroxivitamin D is decreased [**42**,**43**].

## Discussion 

The major motivation of relating VD deficiency to primary HPT and the need to analyze the outcome after PTx in this situation is related to new data regarding its high prevalence in heterogeneous populations, including patients with parathyroid disorders [**44**,**45**].Separately from the PTH excess, low VDmight act as a confounding factor knowing its correlation to skeleton health and the underlying pathways of many others non-bone morbidities [**46**-**48**].A connection between low VD and cardio-metabolic impairment, such as arterial hypertension, obesity, or type 2 diabetes mellitus has been described although the direct cause-effect relationship is not completely understood yet [**49**-**52**]. This aspect might interfere with cardiovascular alterations, which are already presented in primary HPT [**53**-**55**].Other observations are related to the VD implications on immune system, which interfere with anesthesia practice and a longer hospitalization, and associated costs with a higher rate of complications and even mortality have been found in patients with chronic conditions requiring surgery if their VD level was low [**56**].Critically ill patients commonly lack an adequate VD, and this may become a hidden enemy of a good outcome and new protocols of case finding strategies as well as nutritional improvement are necessary [**57**,**58**].

## Conclusion

The growing evidence regarding the high prevalence of hypovitaminosis D and the early detection of primary HPT requires a particular attention to the association of these two disorders, which may be incidental but some common pathogenic links are displayed. VD deficiency, otherwise a commonly recognized condition, is associated with larger parathyroid adenomas and higher levels of PTH before and after surgery for primary HPT. VD supplements are safe under some serum calcium cutoffs and offer a better outcome after PTx. However, primary HPT is cured by surgery and, if the indication is well established, this should not be delayed too long to replace VD. A close following of 25-hydroxivitamin D represents one of the keys for a good outcome in the field of parathyroid surgery. 

**Conflict of interest**

The authors have nothing to declare.

**Acknowledgement**

None.
